# Transition from post‐capillary pulmonary hypertension to combined pre‐ and post‐capillary pulmonary hypertension in swine: a key role for endothelin

**DOI:** 10.1113/JP275987

**Published:** 2018-06-21

**Authors:** Richard W. B. van Duin, Kelly Stam, Zongye Cai, André Uitterdijk, Ana Garcia‐Alvarez, Borja Ibanez, A. H. Jan Danser, Irwin K. M. Reiss, Dirk J. Duncker, Daphne Merkus

**Affiliations:** ^1^ Division of Experimental Cardiology Department of Cardiology Thoraxcenter Erasmus MC Rotterdam The Netherlands; ^2^ Centro Nacional de Investigaciones Cardiovasculares Carlos III (CNIC) Madrid Spain; ^3^ Hospital Clinic of Barcelona IDIBAPS Barcelona Spain; ^4^ IIS‐Fundación Jiménez Díaz University Hospital Madrid Spain; ^5^ CIBERCV Madrid Spain; ^6^ Department of Pharmacology Erasmus MC Rotterdam The Netherlands; ^7^ Pediatrics / Neonatology Erasmus MC – Sophia Children's Hospital Rotterdam The Netherlands

**Keywords:** pulmonary hypertension, endothelin, translational study

## Abstract

**Key points:**

- Passive, isolated post‐capillary pulmonary hypertension (PH) secondary to left heart disease may progress to combined pre‐ and post‐capillary or ‘active’ PH
- This ‘activation’ of post‐capillary PH significantly increases morbidity and mortality, and is still incompletely understood.
- In this study, pulmonary vein banding gradually produced post‐capillary PH with structural and functional microvascular remodelling in swine.
- Ten weeks after banding, the pulmonary endothelin pathway was upregulated, likely contributing to pre‐capillary aspects in the initially isolated post‐capillary PH.
- Inhibition of the endothelin pathway could potentially stop the progression of early stage post‐capillary PH.

**Abstract:**

Passive, isolated post‐capillary pulmonary hypertension (IpcPH) secondary to left heart disease may progress to combined pre‐ and post‐capillary or ‘active’ PH (CpcPH) characterized by chronic pulmonary vascular constriction and remodelling. The mechanisms underlying this ‘activation’ of passive pulmonary hypertension (PH) remain incompletely understood. Here we investigated the role of the vasoconstrictor endothelin‐1 (ET) in the progression from IpcPH to CpcPH in a swine model for post‐capillary PH. Swine underwent pulmonary vein banding (PVB; *n* = 7) or sham‐surgery (Sham; *n* = 6) and were chronically instrumented 4 weeks later. Haemodynamics were assessed for 8 weeks, at rest and during exercise, before and after administration of the ET receptor antagonist tezosentan. After sacrifice, the pulmonary vasculature was investigated by histology, RT‐qPCR and myograph experiments. Pulmonary arterial pressure and resistance increased significantly over time. mRNA expression of prepro‐endothelin‐1 and endothelin converting enzyme‐1 in the lung was increased, while ET_A_ expression was unchanged and ET_B_ expression was downregulated. This was associated with increased plasma ET levels from week 10 onward and a more pronounced vasodilatation to *in vivo* administration of tezosentan at rest and during exercise. Myograph experiments showed decreased endothelium‐dependent vasodilatation to Substance P and increased vasoconstriction to KCl in PVB swine consistent with increased muscularization observed with histology. Moreover, maximal vasoconstriction to ET was increased whereas ET sensitivity was decreased. In conclusion, PVB swine gradually developed PH with structural and functional vascular remodelling. From week 10 onward, the pulmonary ET pathway was upregulated, likely contributing to pre‐capillary activation of the initially isolated post‐capillary PH. Inhibition of the ET pathway could thus potentially provide a pharmacotherapeutic target for early stage post‐capillary PH.

## Key points

- Passive, isolated post‐capillary pulmonary hypertension (PH) secondary to left heart disease may progress to combined pre‐ and post‐capillary or ‘active’ PH
- This ‘activation’ of post‐capillary PH significantly increases morbidity and mortality, and is still incompletely understood.
- In this study, pulmonary vein banding gradually produced post‐capillary PH with structural and functional microvascular remodelling in swine.
- Ten weeks after banding, the pulmonary endothelin pathway was upregulated, likely contributing to pre‐capillary aspects in the initially isolated post‐capillary PH.
- Inhibition of the endothelin pathway could potentially stop the progression of early stage post‐capillary PH.

## Introduction

Pulmonary hypertension (PH) is a pathophysiological disorder that is defined by a mean pulmonary arterial pressure (mPAP) of >25 mmHg at rest (Galie *et al*. 2016). While PH has many different aetiologies, in 65–80% of all cases PH is due to left heart disease, i.e. WHO classification group II (Galie *et al*. 2016; Rosenkranz *et al*. 2016). In this group of PH, left heart failure (HF), valvular disease, inflow‐/outflow‐tract obstructions or congenital or acquired pulmonary vein stenosis causes an upstream pressure increase in the pulmonary vasculature. Initially, this isolated post‐capillary PH (IpcPH) is purely passive, i.e. a direct consequence of the increased pulmonary venous pressure. However, when left untreated, IpcPH has the potential to progress to active, combined pre‐ and post‐capillary PH (CpcPH), a chronic progressive disease characterized by marked pulmonary vasoconstriction and vascular remodelling (Miller *et al*. 2013; Gerges *et al*. 2015; Vanderpool & Naeije, 2015) with a worse prognosis than IpcPH (Tatebe *et al*. 2012). While IpcPH can be treated by correcting only the underlying condition (Galie *et al*. 2016), CpcPH requires treatment of pulmonary vascular remodelling as well as the primary disease, as treatment of the primary disease alone is no longer able to arrest the progression of CpcPH (Lundgren & Radegran, 2014). Hence, understanding the time course of the transition from IpcPH to CpcPH as well as its underlying mechanism is essential. IpcPH is defined as a mPAP of >25 mmHg, with a pulmonary capillary wedge pressure (PCWP) >15 mmHg and a diastolic pressure gradient (difference between diastolic PAP and PCWP) <7 mmHg and/or a pulmonary vascular resistance (PVR) ≤3 Wood units (WU), while CpcPH is defined as a diastolic pressure gradient of ≥7 mmHg and/or a PVR >3 WU (Naeije & D'Alto, 2016). Discriminating IpcPH from CpcPH requires right heart catheterization to measure PCWP. Such an invasive procedure is not suitable for a longitudinal clinical study. In contrast, pre‐clinical animal models for post‐capillary PH are available but most haemodynamic data are acquired at only one or two time points, often under anaesthesia (Endo *et al*. 2002; Dai *et al*. 2004; Garcia‐Alvarez *et al*. 2013; Pereda *et al*. 2014). In PH, not only vasoconstriction but potentially also vascular remodelling may be due to an imbalance between vasoconstrictors and vasodilators, which may involve downregulation of the nitric oxide and prostacyclin pathways, and upregulation of the endothelin (ET) pathway (Moraes *et al*. 2000; Budhiraja *et al*. 2004). Although changes in these pathways have been well characterized in both experimental (Dai *et al*. 2004; Houweling *et al*. 2006; Merkus *et al*. 2007, 2008; van Duin *et al*. 2018) and clinical (Cody *et al*. 1992; Giaid *et al*. 1993; Porter *et al*. 1993; Cooper *et al*. 1998; Meoli *et al*. 2018) studies of PH, the contribution of alterations in these pathways to the transition from IpcPH to CpcPH remains incompletely understood. Here, we hypothesize that the transition from IpcPH to CpcPH is mediated, at least in part, by increased activity of the ET pathway. To test this hypothesis, we investigated the progression of PH using chronically instrumented swine (De Wijs‐Meijler *et al*. 2016), in a recently developed swine model of group II PH by pulmonary vein banding (PVB) (Garcia‐Alvarez *et al*. 2013; Aguero *et al*. 2014; Pereda *et al*. 2014). Repeated measurements of pulmonary haemodynamics in awake swine were performed between 5 and 12 weeks after PVB, during the progression of PH while evaluating the activity of the ET pathway. Since exercise testing allows detection of perturbations in cardiopulmonary function that may not be apparent under quiet resting conditions, and thus facilitates the assessment of disease severity (Keusch *et al*. 2014), we performed measurements both at rest and during graded treadmill exercise.

## Methods

### Ethical approval

Studies were performed in accordance with the Guiding Principles in the Care and Use of Laboratory Animals as approved by the Council of the American Physiological Society, and with approval of the Animal Care Committee of the Erasmus University Medical Centre (EMC3158, 109‐13‐09). The authors understand the ethical principles under which *The Journal of Physiology* operates and this work complies with the animal ethics checklist outlined by Grundy (2015).

### Sample size calculations

Experiments were designed to minimize the number of animals used. To calculate minimal sample size, a power analysis was performed using specialized software (G*Power 3.0) (Faul *et al*. 2007). Assuming a similar increase in PVR at 3 months post‐PVB as in a previous study (Pereda *et al*. 2014), and a similar decrease in PVR after administration of tezosentan as reported previously (Merkus *et al*. 2007), a two‐tailed approach with assumed α‐error probability of 0.5 and a power of 80% led to a sample size of *n* = 5 per experimental group. Taking into consideration a drop‐out of 25% as a result of (post‐)surgical complications, and a 10% drop‐out due to malfunction of the catheters, target group size was set to *n* = 8. Thirteen animals completed the protocol and are included in the final analyses.

### Study design

Fifteen crossbred Landrace × Yorkshire swine of either sex (9 ± 1 kg) underwent non‐restrictive inferior pulmonary vein banding (*n* = 9) or sham operation (*n* = 6). Four weeks later, all 14 surviving animals (22 ± 1 kg) were chronically instrumented to enable weekly monitoring of haemodynamics and blood gases in animals in the awake state. In the following 8 weeks, animals performed bi‐weekly exercise experiments under control conditions and after administration of an ET_A_/ET_B_ receptor antagonist. After 12 weeks, the surviving 13 animals (62 ± 2 kg) were sacrificed and tissues were harvested.

### Pulmonary vein banding

Swine underwent banding of the inferior venous confluence as described by Pereda *et al*. (2014). Briefly, swine were sedated with an intramuscular (i.m.) injection of tiletamine/zolazepam (5 mg kg^−1^; Virbac, Barneveld, The Netherlands), xylazine, (2.25 mg kg^−1^; AST Pharma, Oudewater, The Netherlands) and atropine (1 mg), anaesthetized with intravenous (i.v.) bolus administration of thiopental (10 mg kg^−1^; Rotexmedica, Trittau, Germany), intubated and ventilated with a mixture of O_2_ and N_2_ (1:2) to which isoflurane (2% v/v; Pharmachemie, Haarlem, The Netherlands) was added. Animals received antibiotic prophylaxis prior to the procedure (0.75 ml Depomycine, 200,000 IU ml^−1^ procainebenzylpenicilline, 200 mg ml^−1^ dihydrostreptomycine; Intervet Schering‐Plough, Boxmeer, The Netherlands). Under sterile conditions, the chest was opened via the fifth right intercostal space and the inferior venous confluence, draining both inferior pulmonary lobes, was exposed and separated from surrounding lung tissue using blunt dissection. A surgical loop (Braun Medical Inc., Bethlehem, PA, USA) was passed around the vein near the left atrium and secured at the resting diameter with a silk suture. The ribs were secured using non‐absorbable USP6 braided polyester (Ø 0.8 mm) and the wound was closed in layers using silk sutures. Anaesthesia was terminated and animals were extubated once spontaneous ventilation was restored. Animals received analgesia (0.3 mg buprenorphine i.m.; Indivior, Slough, UK) and a fentanyl slow‐release patch (6 μg h^−1^, 48 h) and were transferred back to the animal facilities once awake. Six swine (8 ± 1 kg) underwent a sham procedure, performed exactly as described above where the inferior venous confluence was exposed and dissected free, but not banded. In the PVB group, one animal died immediately after the banding procedure upon commencement of spontaneous breathing, due to acute pulmonary oedema. Taking humane endpoints into account, one animal was prematurely euthanized 6 weeks after banding due to severe HF. Data from these animals have been excluded from analysis. In the sham group, all animals survived until the end of follow‐up, resulting in group sizes of *n* = 7 for PVB and *n* = 6 for sham.

### Chronic instrumentation

Four weeks after PVB or sham procedure, swine were anaesthetized and ventilated as described above and chronically instrumented as described previously (De Wijs‐Meijler *et al*. 2016). Briefly, under sterile conditions, the chest and pericardium were opened via the fourth left intercostal space and fluid‐filled polyvinylchloride catheters (Braun Medical Inc.) were inserted into the aortic arch, the pulmonary artery, the right ventricle and the left atrium for blood pressure measurement and blood sampling. A transit time flowprobe (Transonic Systems Inc., Ithaca, NY, USA) was positioned around the ascending aorta for measurement of cardiac output. Catheters were tunnelled to the back and the chest was closed. Animals were allowed to recover, receiving a single shot of buprenorphine i.m. (0.3 mg) and a fentanyl slow‐release patch (12 μg h^−1^, 48 h) for analgesia and antibiotic prophylaxis consisting of amoxicillin (25 mg kg^−1^ i.v.; Centrafarm B.V. Etten‐Leur, The Netherlands) and gentamycin (5 mg kg^−1^ i.v.; Eurovet, Bladel, The Netherlands) for seven consecutive days post‐surgery. Once fully awake, animals were transferred back to the animal facility.

### Experimental protocols

Resting haemodynamic measurements were obtained each week, while exercise studies were performed 6, 8, 10 and 12 weeks following the PVB procedure. Swine were transferred to a customized, motor‐driven treadmill and the fluid‐filled catheters were connected to pressure transducers (Combitrans pressure transducers, Braun, Melsungen, Germany). Transducers and flowprobe were connected to amplifiers and haemodynamics were recorded at rest and during four‐stage incremental treadmill exercise (1–4 km h^−1^, 3 min per speed). Heart rate, cardiac output and left atrial, aortic, right ventricular and pulmonary arterial blood pressures were continuously recorded and blood samples were collected during the last 60 s of each 3 min exercise stage, at a time when haemodynamics had reached a steady state. After 60 min of rest, at a time when haemodynamics had returned to baseline, the ET_A_/ET_B_ receptor antagonist tezosentan (gift from Dr Clozel, Actelion Pharmaceuticals Ltd) was administered by an infusion of 300 μg kg^−1^ min^−1^ i.v. After 10 min of infusion the exercise protocol was repeated, with continuous infusion of 100 μg kg^−1^ min^−1^ i.v. during the entire protocol. Due to recurrent crippling, one PVB animal and one sham animal did not participate in all exercise experiments, reducing the number of animals to five in the sham group and six in the PVB group. Moreover, due to technical failure, flow probe data of one PVB animal were not available, reducing the number of animals to five for flow‐related parameters (cardiac output, stroke volume, systemic and pulmonary vascular resistance, body oxygen consumption).

### Blood gas measurements

Arterial and mixed venous blood samples were kept in iced syringes before being processed by a blood gas analyser (ABL 800, Radiometer, Denmark). Measurements included $P_{o_{2}}$ (mmHg), $P_{co_{2}}$ (mmHg), oxygen saturation(%), lactate and haemoglobin (grams per decilitre). Body O_2_ consumption ($B{\dot{V}}_{O_{2}}$) was calculated as the product of cardiac output and the difference in O_2_ content between arterial and mixed venous blood, where blood O_2_ content (μmol ml^−1^) was computed as (Hb × 0.621 O_2_ saturation) + (0.00131$P_{O_{2}}$) (Duncker *et al*. 2000).

### Data analysis

Digital recording and off‐line haemodynamic analyses were described previously (Haitsma *et al*. 2002). CO was corrected for body weight (cardiac index, CI). Total pulmonary vascular resistance index (tPVRi) and systemic vascular resistance index (SVRi) were calculated as mPAP/CI and mean arterial pressure MAP/CI respectively. Stroke volume index was computed as CI divided by heart rate and body oxygen consumption index ($B{\dot{V}}_{O_{2}}i$) as B${\dot{V}}_{O_{2}}$ divided by body weight.

### Sacrifice

Upon completion of follow‐up, i.e. 12 weeks after banding or sham surgery, animals were sedated with i.v. tiletamine/zolazepam (5 mg kg^−1^), xylazine, (2.25 mg kg^−1^) and atropine (1 mg) and anaesthetized with pentobarbital sodium (i.v. 6–12 mg kg^−1^ h^−1^). An endotracheal tube was placed, and animals were ventilated with a mixture of O_2_ and N_2_ (1:2). Following sternotomy, the heart was arrested and immediately excised together with the lungs. Parts of the upper and lower lobe of the right lung were snap frozen in liquid nitrogen for molecular analyses, or processed for histology. Other parts of the upper and lower lobe were placed in cold Krebs buffer for dissection of pulmonary small arteries for wire‐myograph experiments. The left and right ventricle of the heart were weighed separately to assess right ventricular hypertrophy.

### Plasma ET measurements

Two‐weekly blood samples were collected in EDTA‐coated tubes, centrifuged for 10 min at 1460 *g* and 4°C and plasma was subsequently aliquoted and cryopreserved at −80°C until analysis. For measurement of plasma ET levels, an ET enzyme‐linked immunosorbent assay (ELISA)‐kit was used according to the manufacturer's protocol (Enzo Life Sciences International Inc., Farmingdale, NY, USA).

### Real‐time quantitative PCR of lung tissue

For measurement of prepro‐ET‐1 (PPET), ET converting enzyme‐1 (ECE), ET receptor A (ET_A_) and ET receptor B (ET_B_) mRNA levels, lung tissue was snap frozen in liquid nitrogen after excision. Small pieces of tissue (<30 mg) were homogenized by adding RLT lysis buffer (Qiagen, Venlo, The Netherlands) and 2‐mercaptoethanol (Sigma‐Aldrich, Zwijndrecht, The Netherlands) using a homogenizer. After a proteinase K (Invitrogen, Breda, The Netherlands) treatment at 55°C for 10 min, total RNA was isolated using RNeasy Fibrous Tissue Mini Kit (Qiagen). RNA was eluted in RNase‐free water and the concentration was determined using a NanoDrop (NanoDrop1000, Thermo Fisher Scientific, Bleiswijk, The Netherlands). RNA integrity was confirmed by Bioanalyzer (2100 Bioanalyzer, Agilent, Santa Clara, CA, USA). cDNA was synthesized from 500 ng of total RNA with SensiFAST cDNA Synthesis Kit (Bioline, London, UK). RT‐qPCR (CFX‐96, Bio‐Rad, Hercules, CA, USA) was performed with SensiFAST SYBR & Fluorescein Kit (Bioline). Target gene mRNA levels were normalized against β‐actin, glyceraldehyde‐3‐phosphate dehydrogenase (GADPH) and cyclophilin using the CFX manager software (Bio‐Rad). Relative gene expression data were calculated using the ΔΔ*C*_t_ method. Expression relative to sham was calculated by dividing individual expressions of PVB as well as sham animals by mean sham expression.

### Wire myograph experiments

*In vitro* pulmonary vessel experiments were performed as previously described (Mulvany & Halpern, 1977; Zhou *et al*. 2014). In short, pulmonary small arteries were dissected and stored overnight in oxygenated (95% O_2_–5% CO_2_) Krebs bicarbonate solution (composition in mm: 118 NaCl, 4.7 KCl, 2.5 CaCl_2_, 1.2 MgSO_4_, 1.2 KH_2_PO_4_, 25 NaHCO_3_, glucose 8.3; pH 7.4) at 4°C. The next day, 2 mm segments were obtained and mounted on wire myographs in separate organ baths with oxygenated Krebs–bicarbonate solution at 37°C. Following a stabilization period of 30 min, internal vessel diameter was set to a tension equivalent of 0.9 times the estimated diameter at 20 mmHg effective transmural force. After pre‐constriction using 100 nm of the synthetic thromboxane analogue U46619, endothelial integrity was ascertained by administration of the endothelium‐dependent vasodilator substance P (10 nm). Maximal constriction was tested by exposure of the vessels to 100 mm KCl. After 30 min of stabilization in fresh buffer solution, vessels were incubated with either no blocker, the ET_A_ blocker BQ123 (10^−6^ m) or the ET_B_ blocker BQ788 (10^−8^ m). To test contraction to ET, vessels were subjected to incremental dosages from 10^−10^ to 3 × 10^−7^ m ET. Constriction to ET was calculated as percentage of maximal constriction to 100 mm KCl. Data were analysed using designated software (Labchart 8.0, ADInstruments, Sydney, Australia), and concentration–response curves were created using Prism (version 5.0, GraphPad Software, Inc., La Jolla, CA, USA) and StatView (version 5.0, SAS Institute, Cary, NC, USA).

### Histology

Tissue from the upper and lower lobe of the right lung was fixed in 3.5–4% buffered formaldehyde for at least 48 h and subsequently dehydrated in incremental alcohol solutions, xylene and finally embedded in paraffin wax. Transverse sections (4.5 μm) were cut, using a microtome, and mounted on glass slides. Resorcin–Fuchsin–von Gieson (RF) staining was performed to discriminate the internal and external elastic lamina in small pulmonary arteries. Using the Hamamatsu NanoZoomer Digital Pathology (NDP) slide scanner (Hamamatsu Nanozoomer 2.0HT, Hamamatsu Photonics K.K., Hamamatsu City, Japan), whole section images were obtained. Morphometric measurements of pulmonary small arteries were performed using NDP viewer (Hamamatsu). Both internal and external elastic lamina areas were measured and assuming circularity of the vessels, inner and outer radius were calculated as *r* = √(area/π). Wall‐to‐lumen ratio was calculated as (outer − inner radius)/inner radius, and relative lumen area as outer/inner area. To ensure that pulmonary veins were excluded from analysis, vessels in close proximity to the intersegmental septae were excluded from analysis. Only transversely cut vessels with an outer diameter of 50–150 μm were analysed.

### Statistical analysis

SPSS Statistics (version 21.0, IBM Corp., Armonk, NY, USA) was used for statistical analysis. Statistical analysis was performed using Student's *t* test, or one‐way or two‐way ANOVA for repeated measures, followed by *post hoc* testing with the Bonferroni test, when appropriate. Concentration–response curves were analysed by regression analyses using Prism (version 5) and Statview (Version 5.0). Statistical significance was accepted when *P *≤ 0.05 (two‐tailed). Data are presented as means ± SEM. Individual data below the (1st quartile – 1.5 × interquartile range) and above the (3rd quartile + 1.5 × interquartile range) thresholds were considered outliers, and were excluded from statistical analyses.

## Results

### Induction of pulmonary hypertension

**Figure 1:**
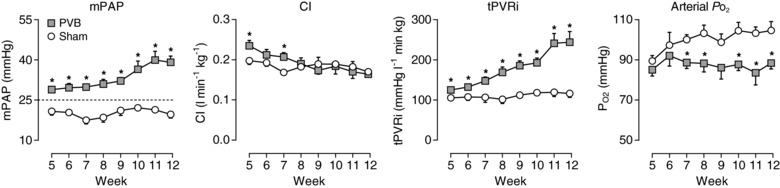
Progression of mean pulmonary artery pressure (mPAP), cardiac index (CI), total pulmonary vascular resistance index (tPVRi) and arterial partial oxygen pressure ($P_{O_{2}}$) at rest over time Values are means ± SEM. Dotted line represents clinical threshold for PH. ^*^ *P* ≤ 0.05 *vs*. sham by two‐way ANOVA for repeated measures with Bonferroni posthoc test. PVB, *n* = 7 for mPAP, *n* = 6 for CI and tPVRi; Sham, *n* = 6. PVB, pulmonary vein banding.

Already after 5 weeks, pulmonary vein banding resulted in an increased tPVRi of 129 ± 6 *vs*. 105 ± 5 mmHg l^−1^ min kg in sham animals (*P* ≤ 0.05). This increased resistance was reflected in pulmonary hypertension: mPAP = 29 ± 1 *vs*. 21 ± 1 mmHg in sham (*P* ≤ 0.01). Both tPVRi and mPAP increased over time, reflecting the progressive nature of PH (Fig. 1; week 12 tPVRi: 255 ± 31 *vs*. 116 ± 10 mmHg l^−1^ min kg; mPAP: 39 ± 1 *vs*. 20 ± 2 mmHg; both *P* ≤ 0.01). Resting systemic haemodynamics remained unchanged as compared to sham group (Table 1) and cardiac index, while transiently higher in the PVB group at week 5 and 7, remained essentially similar in both groups (Fig. 1). PVB resulted in a markedly lower arterial $P_{O_{2}}$ from 7 weeks after banding, which persisted until follow‐up at 12 weeks (Table 2; 87 ± 4 *vs*. 101 ± 4 mmHg; *P* ≤ 0.05) and arterial oxygen saturation tended to be lower at 12 weeks (96 ± 1 *vs*. 98 ± 1%; *P *= 0.06). Mixed venous $P_{O_{2}}$ and oxygen saturation, arterial and mixed venous $P_{CO_{2}}$ and haemoglobin were similar between PVB and sham animals.

**Table 1:** Haemodynamics at rest and during exercise before and after administration of tezosentan

					Exercise (km h^−1^)	
	Group	Treatment	*n*	Rest	2	4
HR (bpm)	Sham	Control	5	133 ± 10	195 ± 7*	256 ± 9*
		Tezosentan	5	160 ± 12‡	203 ± 13*	263 ± 11*
	PVB	Control	6	131 ± 7	173 ± 6* †	198 ± 6* †
		Tezosentan	6	145 ± 3	180 ± 5*	208 ± 9* †
MAP (mmHg)	Sham	Control	5	96 ± 3	101 ± 2	103 ± 3
		Tezo	5	86 ± 4	88 ± 1‡	95 ± 2‡
	PVB	Control	6	86 ± 5	86 ± 4†	84 ± 6†
		Tezosentan	6	73 ± 3‡ †	77 ± 3‡ †	78 ± 4†
LAP (mmHg)	Sham	Control	5	3.0 ± 0.5	6.8 ± 0.9	9.0 ± 0.3*
		Tezosentan	5	6.3 ± 2.9	8.8 ± 1.3	13.3 ± 1.0‡
	PVB	Control	6	5.7 ± 1.4	9.4 ± 1.2*	12.8 ± 1.9*
		Tezosentan	6	4.4 ± 1.7	7.8 ± 1.3* ‡	13.7 ± 2.8*
mPAP (mmHg)	Sham	Control	5	20 ± 1	30 ± 3*	35 ± 3*
		Tezosentan	5	20 ± 2	28 ± 3*	35 ± 3*
	PVB	Control	6	35 ± 2†	55 ± 4* †	59 ± 3* †
		Tezosentan	6	33 ± 3†	48 ± 4* ‡ †	57 ± 3* †
CI (l min^−1^ kg^−1^)	Sham	Control	5	0.17 ± 0.01	0.24 ± 0.02*	0.28 ± 0.02*
		Tezosentan	5	0.19 ± 0.01	0.24 ± 0.01*	0.28 ± 0.02*
	PVB	Control	5	0.17 ± 0.01	0.22 ± 0.01*	0.23 ± 0.01*
		Tezosentan	5	0.18 ± 0.01	0.22 ± 0.01*	0.23 ± 0.01*
SVi (ml kg^−1^)	Sham	Control	5	1.29 ± 0.13	1.23 ± 0.12	1.09 ± 0.11
		Tezosentan	5	1.23 ± 0.12	1.20 ± 0.12	1.09 ± 0.10
	PVB	Control	5	1.28 ± 0.07	1.27 ± 0.08	1.20 ± 0.07
		Tezosentan	5	1.25 ± 0.09	1.24 ± 0.08	1.14 ± 0.07
SVRi (mmHg l^−1^ min kg)	Sham	Control	5	575 ± 26	437 ± 41*	381 ± 28*
		Tezosentan	5	453 ± 29‡	375 ± 22	343 ± 29
	PVB	Control	5	522 ± 44	405 ± 33*	364 ± 38*
		Tezosentan	5	411 ± 44‡	351 ± 29‡	334 ± 25*

Data are means ± SEM. Haemodynamics obtained 12 weeks after banding. ^*^ *P* ≤ 0.05 *vs*. rest; ^†^ *P* ≤ 0.05 *vs*. corresponding sham; ^‡^ *P* ≤ 0.05 *vs*. corresponding control experiment. CI, cardiac index; HR, heart rate; LAP, mean left atrial pressure; MAP, mean arterial pressure; mPAP, mean pulmonary arterial pressure; SVi, stroke volume index (CI/HR); SVRi, systemic vascular resistance index (MAP/CI).

**Table 2:** Blood gas analyses at rest and during exercise before and after administration of tezosentan

					Exercise (km h^−1^)	
	Group	Treatment	*n*	Rest	2	4
$P_{O_{2}}$ art (mmHg)	Sham	Control	5	101 ± 4	89 ± 4*	90 ± 5*
		Tezosentan	5	98 ± 2	95 ± 6	90 ± 5
	PVB	Control	6	87 ± 4†	69 ± 4* †	65 ± 4* †
		Tezosentan	6	82 ± 3†	67 ± 4* †	63 ± 4* †
$P_{O_{2}}$ mv (mmHg)	Sham	Control	5	42 ± 1	35 ± 1*	30 ± 1*
		Tezo	5	46 ± 1‡	38 ± 2*	30 ± 2*
	PVB	Control	6	41 ± 2	31 ± 2*	27 ± 2*
		Tezosentan	6	42 ± 2	34 ± 2*	28 ± 2*
$S_{aO_{2}}$ art (%)	Sham	Control	5	98 ± 1	95 ± 1*	96 ± 1
		Tezosentan	5	97 ± 1	96 ± 1	96 ± 1
	PVB	Control	6	96 ± 1	90 ± 3	88 ± 2* †
		Tezosentan	6	95 ± 1	89 ± 2* †	86 ± 3* †
$S_{aO_{2}}$ mv (%)	Sham	Control	5	55 ± 1	40 ± 2*	31 ± 3*
		Tezosentan	5	63 ± 2‡	48 ± 5* ‡	31 ± 4*
	PVB	Control	6	56 ± 2	35 ± 3*	25 ± 3*
		Tezosentan	6	58 ± 3	41 ± 2*	28 ± 4*
$P_{CO_{2}}$ art (mmHg)	Sham	Control	5	40 ± 2	43 ± 2*	37 ± 3
		Tezosentan	5	41 ± 2	39 ± 2‡	35 ± 2*
	PVB	Control	6	38 ± 3	37 ± 1†	36 ± 2
		Tezosentan	6	40 ± 2	39 ± 2	36 ± 2
$P_{CO_{2}}$ mv (mmHg)	Sham	Control	5	49 ± 1	52 ± 2	53 ± 3
		Tezosentan	5	47 ± 2	49 ± 2	48 ± 2
	PVB	Control	6	48 ± 2	47 ± 2	49 ± 3
		Tezosentan	6	44 ± 3	43 ± 2	44 ± 3
Hb (g dl^−1^)	Sham	Control	5	9.4 ± 0.4	11.0 ± 0.3*	11.2 ± 0.3*
		Tezosentan	5	9.9 ± 0.6	10.5 ± 0.2	10.9 ± 0.3
	PVB	Control	6	9.4 ± 0.7	10.0 ± 0.5	10.4 ± 0.8
		Tezosentan	6	9.5 ± 0.6	10.1 ± 0.6	10.6 ± 0.6
Lac art (mmol l^−1^)	Sham	Control	5	0.78 ± 0.07	0.88 ± 0.07*	1.86 ± 0.28*
		Tezosentan	5	0.72 ± 0.06	0.78 ± 0.05	2.46 ± 0.53*
	PVB	Control	6	0.87 ± 0.04	0.80 ± 0.04	1.77 ± 0.33*
		Tezosentan	6	0.83 ± 0.05	0.78 ± 0.05	1.95 ± 0.52
$B{\dot{V}}_{O_{2}}i$ (mmol min^−1^ kg^−1^)	Sham	Control	5	0.43 ± 0.03	0.92 ± 0.08*	1.26 ± 0.09*
		Tezosentan	5	0.40 ± 0.03	0.75 ± 0.05*	1.25 ± 0.06*
	PVB	Control	6	0.42 ± 0.04	0.75 ± 0.03*	1.00 ± 0.02* †
		Tezosentan	6	0.42 ± 0.05	0.65 ± 0.02*	0.92 ± 0.01* ‡ †

Data are means ± SEM. Blood gas analyses obtained 12 weeks after banding. ^*^ *P* ≤ 0.05 *vs*. rest; ^†^ *P* ≤ 0.05 *vs*. corresponding sham; ^‡^ *P* ≤ 0.05 *vs*. corresponding control. art, arterial; $B{\dot{V}}_{O_{2}}i$, body oxygen consumption index (CI × arterio‐venous oxygen difference); Hb, haemoglobin; Lac, lactate; mv, mixed venous; $P_{O_{2}}$, partial oxygen pressure; $P_{CO_{2}}$, partial carbon dioxide pressure; $S_{aO_{2}}$, oxygen saturation.

### Right ventricular remodelling

**Figure 2:**
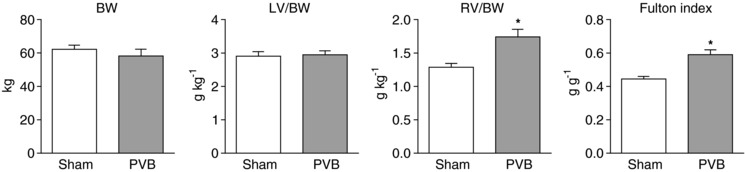
Right ventricular mass increased in PVB animals (*n* = 7) as compared to sham animals (*n* = 6) Values are means ± SEM. ^*^ *P* ≤ 0.05 *vs*. sham by unpaired *t* test (two‐sided). BW, body weight; LV, left ventricle; RV, right ventricle.

At sacrifice, body weight and left ventricle to body weight ratio were similar between groups (Fig. 2). Both the right ventricle to body weight ratio (1.74 ± 0.11 *vs*. 1.29 ± 0.06 g kg^−1^) and the Fulton index, calculated as ratio of right ventricular to left ventricular weight (0.59 ± 0.03 *vs*. 0.44 ± 0.01 g g^−1^) were increased, indicating right ventricular hypertrophy.

### Response to exercise

**Figure 3:**
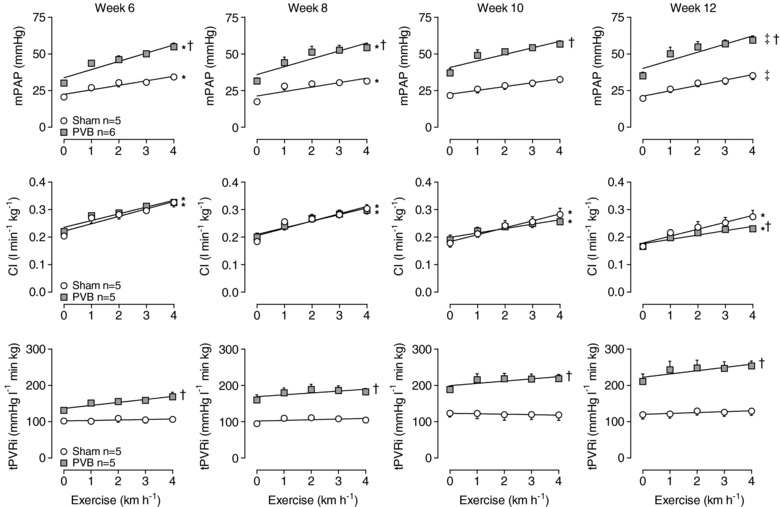
Haemodynamic response to exercise in week 6, 8, 10 and 12 Values are means ± SEM. ^*^ *P* ≤ 0.05 effect exercise; ^†^ *P* ≤ 0.05 *vs*. sham; ^‡^ *P *= 0.07 effect of exercise two‐way ANOVA for repeated measures. CI, cardiac index; mPAP, mean pulmonary arterial pressure; PVB, pulmonary vein banding; tPVRi, total pulmonary vascular resistance index.

At all time points during follow‐up, exercise up to 4 km h^−1^ produced an increase in mPAP in sham animals that was the consequence of an increase in LAP in combination with an increase in CI, while tPVRi remained essentially unchanged (Fig. 3). Arterial O_2_‐saturation remained virtually unaffected in sham animals, but decreased significantly with exercise in PVB animals (Table 2). From week 6 until week 10, the exercise‐induced increases in LAP (not shown) and CI (Fig. 3) were similar in PVB and sham animals, and although resting mPAP and tPVRi were higher in PVB as compared to sham, the exercise‐induced increases in mPAP and tPVRi were similar. In week 12, CI was lower during exercise in PVB animals than in sham animals (Fig. 3; CI at 4 km h^−1^: 0.23 ± 0.01 *vs*. 0.28 ± 0.02; *P* ≤ 0.05), which was almost entirely attributed to a lower heart rate in PVB animals compared to sham animals (Table 1; heart rate at 4 km h^−1^: 198 ± 6 *vs*. 256 ± 9 beats min^−1^; *P* ≤ 0.05). Despite this attenuated increase in CI, the exercise‐induced increase in mPAP was similar in PVB and sham animals, as a result of the higher tPVRi in PVB animals.

### Plasma ET levels and effect of ET_A_/ET_B_ blockade by tezosentan

**Figure 4:**
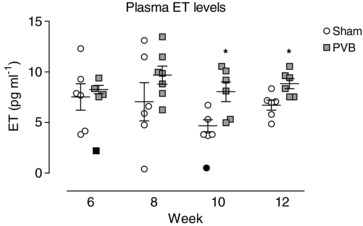
Plasma ET levels over time in PVB and sham animals Values are individual animals and means ± SEM. Data below the (1st quartile – 1.5 × interquartile range) and above the (3rd quartile + 1.5 × interquartile range) thresholds were considered outliers (black symbols), and were excluded from statistical analyses and from mean ± SEM calculations. ^*^ *P* ≤ 0.05 *vs*. sham by unpaired *t* test (two‐sided).

**Figure 5:**
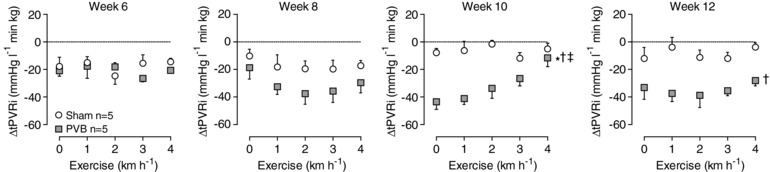
Vasodilatation by ET_A_/ET_B_‐blocker tezosentan had a more pronounced effect on PVB animals in week 10 and 12 ΔtPVRi: delta total pulmonary vascular resistance index: tPVRi with tezosentan – tPVRi without tezosentan Values are means ± SEM. ^*^ *P* ≤ 0.05 effect exercise; ^†^ *P* ≤ 0.05 *vs*. sham; ^‡^ *P* ≤ 0.05 effect exercise PVB *vs*. effect exercise sham by two‐way ANOVA. PVB, *n* = 5; Sham, *n* = 5. PVB, pulmonary vein banding.

Plasma ET levels were similar in PVB and sham animals in weeks 6 and 8 (Fig. 4), and ET_A_/ET_B_ blockade by tezosentan at rest resulted in vasodilatation that was similar in PVB and sham groups in week 6 (Fig. 5); ΔtPVRi: −21 ± 4 *vs*. −18 ± 7 mmHg l^−1^ min kg; not significant) and week 8 (ΔtPVRi: −19 ± 8 *vs*. −10 ± 5 mmHg l^−1^ min kg; not significant). Consistent with the higher plasma ET levels in PVB as compared to sham animals in week 10 (Fig. 4), tezosentan‐induced vasodilatation was significantly more pronounced in PVB than in sham animals at rest at this time point (Fig. 5; resting ΔtPVRi week 10: −43 ± 5 *vs*. −8 ± 3 mmHg l^−1^ min kg; *P* ≤ 0.01) although this effect waned during exercise. In week 12, plasma ET levels remained higher in PVB as compared to sham, and the tezosentan‐induced vasodilatation was now higher in PVB than in sham animals both at rest (Fig. 5; ΔtPVRi: −33 ± 9 *vs*. −12 ± 8 mmHg l^−1^ min kg; *P* ≤ 0.05) and at all exercise intensities.

### Gene expression

**Figure 6:**
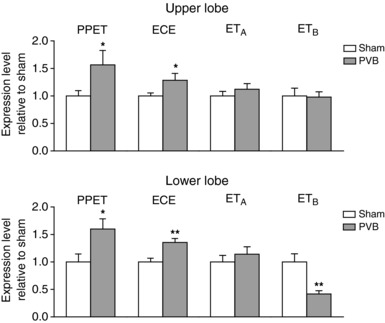
Expression of prepro‐endothelin‐1 (PPET), endothelin converting enzyme 1 (ECE), and receptors ET_A_ and ET_B_ in the upper and lower lobes of PVB and sham animals relative to the corresponding mean expression of sham animals Values are means ± SEM. ^*^ *P* ≤ 0.05, ^**^ *P* ≤ 0.01 by unpaired *t* test (two tailed).

PVB lung tissue showed no change in expression of the ET_A_ receptor while expression of PPET and ECE was upregulated (Fig. 6). In the lower lobes, this was accompanied by a down‐regulation of the ET_B_ receptor, which is the ET‐clearance receptor, while in the upper lobe, expression of the ET_B_ receptor was maintained. Altogether, these data suggest an increased ET production and a decreased ET clearance in the lungs and point towards the lungs as the origin of the increased circulating ET levels.

### Pulmonary microvascular structure and function

**Figure 7:**
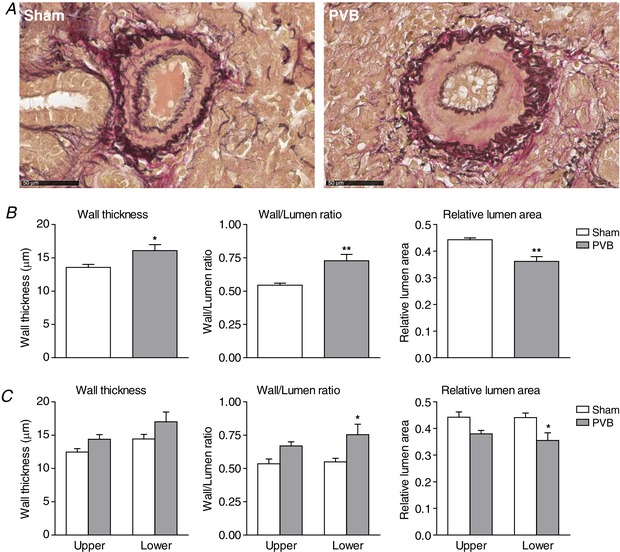
Typical examples (*A*) and histological assessment of small pulmonary arteries (Ø 50–150 μm) showing differences in wall thickness, wall/lumen ratio and relative lumen area between PVB and sham groups (*B*) and between upper and lower lobes of these groups (*C*) Values are means ± SEM. Scale bar: 50 μm. ^*^ *P* ≤ 0.05 *vs*. corresponding sham, ^**^ *P* ≤ 0.01 *vs*. corresponding sham by two‐way ANOVA with the Bonferroni *post hoc* test when appropriate. [Color figure can be viewed at wileyonlinelibrary.com]

Histological assessment of small pulmonary arteries revealed increased wall thickness in PVB as compared to sham (Fig. 7; 16.1 ± 0.9 *vs*. 13.6 ± 0.4 μm; *P* ≤ 0.05), resulting in an increased wall/lumen ratio (0.73 ± 0.05 *vs*. 0.54 ± 0.01; *P* ≤ 0.01) and a decreased relative lumen area (0.36 ± 0.02 *vs*. 0.44 ± 0.01; *P* ≤ 0.01). To elucidate the potential difference between the upper and lower lobes, data were split into four groups, PVB upper lobe, PVB lower lobe, sham upper lobe and sham lower lobe, which showed that differences between PVB and sham groups were essentially similar in both lobes, but reached statistical significance only in the lower lobes (Fig. 7, lower panel).

**Figure 8:**
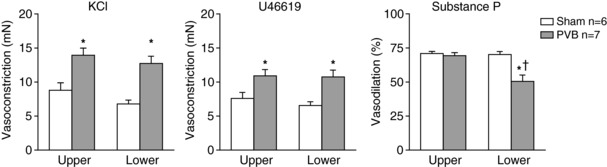
Vasoconstriction response to 100 mm KCl and U46619 was bigger in PVB group compared to sham group, whereas endothelium‐dependent vasodilatation to Substance P was reduced in the lower, but not upper lobes of swine with PVB (C) Values are means ± SEM. KCl and U46619 data are given as absolute values, substance P data as percentage of dilatation after constriction by U46619. Vasodilatation by substance P was impaired in PVB lower lobes, but not in upper lobes. ^*^ *P* ≤ 0.05 *vs*. corresponding sham, ^†^ *P* ≤ 0.05 *vs*. corresponding upper lobe by two‐way ANOVA with the Bonferroni *post hoc* test.

Vasoconstriction in response to KCl (100 mm) and U46619 was increased in vessels isolated from both upper and lower lobes from PVB as compared to sham animals (Fig. 8). These effects reflect the medial hypertrophy of the pulmonary small arteries observed histologically. Endothelial function, as determined by vasodilatation to substance P was similar between upper and lower lobes from sham animals. In PVB animals, the vasodilator response to substance P in the upper lobe was not different from that in the upper lobe of sham swine. However, vasodilatation to substance P was significantly reduced in pulmonary small arteries from the lower lobe of PVB animals (Fig. 8).

**Figure 9:**
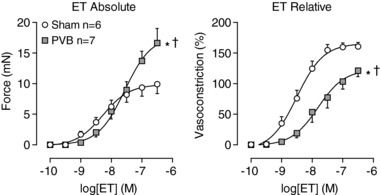
Vasoconstriction by administration of exogenous endothelin‐1 (ET) to isolated small pulmonary arteries Values are means ± SEM. Data are presented as absolute constriction (mN, left panel) and as percentage of maximal vasoconstriction by administration of 100 mm KCl (%, right panel). ^*^ *P* ≤ 0.05 *vs*. sham top, ^†^ *P* ≤ 0.05 *vs*. sham EC_50_ by regression analyses.

Administration of exogenous ET resulted in vasoconstriction in both sham and PVB animals. While absolute maximal vasoconstriction to ET was higher in PVB than in sham animals (16.6 ± 2.4 *vs*. 9.9 ± 1.6 mN; *P* ≤ 0.05), constriction relative to maximal KCl‐induced vasoconstriction was lower in PVB (121 ± 10% *vs*. 161 ± 7%; *P* ≤ 0.01), and sensitivity to ET was reduced in PVB animals (Fig. 9); logEC_50_, 8.3 ± 0.2 *vs*. 7.6 ± 0.2; *P* ≤ 0.05).

**Figure 10:**
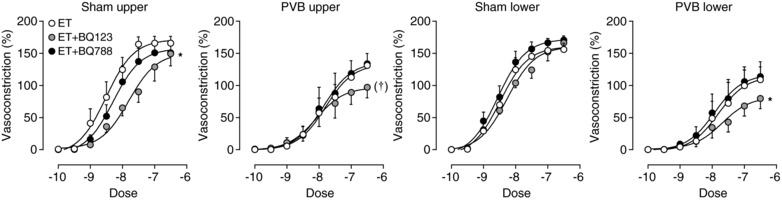
Vasoconstriction by administration of exogenous endothelin‐1 (ET) to isolated small pulmonary arteries of the sham upper and lower lobes, and PVB upper and lower lobes Values are means ± SEM. Vessels were first incubated with either BQ123 for ET_A_ blockade, BQ788 for ET_B_ blockade, or no receptor blocker at all. Data are presented as percentage of maximal vasoconstriction by administration of 100 mm KCl. ^*^ *P* ≤ 0.05 *vs*. ET, ^†^ *P* = 0.06 by regression analyses.

Vessels were incubated with BQ123 or BQ788 to obtain ET_A_ or ET_B_ blockade, and exposed to exogenous ET. The resulting concentration–response curves were compared to those of vessels only exposed to exogenous ET. Under no condition did BQ788 affect the ET concentration–response curve, while BQ123 reduced the ET effects, except in the sham lower lobes (Fig. 10).

## Discussion

The main findings in this study are that (i) non‐restrictive banding of the confluence of the lower pulmonary veins in swine resulted in PH; (ii) within 12 weeks after banding, the initially isolated post‐capillary PH progressed to combined pre‐/post‐capillary PH with structural and functional changes in pre‐capillary pulmonary vessels; (iii) from 10 weeks after banding until sacrifice at 12 weeks, the ET pathway was upregulated, actively contributing to the increased pulmonary vascular resistance.

### Model validation

This swine model for group II PH originated in the group of Ibanez *et al*. (Garcia‐Alvarez *et al*. 2013; Aguero *et al*. 2014; Pereda *et al*. 2014). To date, pulmonary hypertension and RV function have been studied under anaesthesia in this model, and revealed a marked increase in mPAP, PVR and vessel wall thickness that, after 4 months follow‐up, resulted in structural and functional changes in the RV and, in a subset of animals, resulted in RV failure. In the present study, we performed serial measurements of systemic and pulmonary haemodynamics and blood gases in awake swine at rest as well as during graded treadmill exercise. Moreover, the chronic instrumentation allows for serial testing of vasoactive compounds – to study over time the mechanisms that control pulmonary vascular tone – and the serial collection of blood samples. The current study demonstrates that this porcine group II PH model has excellent inter‐study and inter‐group reproducibility, as the present findings at 12 weeks (mPAP and PVR, as well as pulmonary vascular remodelling) are very similar to those of the Ibanez group at three months (Garcia‐Alvarez *et al*. 2013; Pereda *et al*. 2014). The additional chronic instrumentation, and subsequent exercise testing unmasked a mild chronotropic incompetence that has also been shown in PH (Oliveira *et al*. 2017), and has been ascribed to impaired autonomic control (Bristow *et al*. 1992; Wensel *et al*. 2009). The impaired increase in heart rate in PVB *vs*. sham resulted in an attenuated increase in cardiac index and is consistent with the exercise intolerance in group II PH (Caravita *et al*. 2017; Oliveira *et al*. 2017). Clinically, group II PH comprises a variety of different aetiologies, including left HF, valvular disease, inflow‐/outflow‐tract obstructions, and congenital or acquired pulmonary vein stenosis. The pulmonary vein banding swine model is well suited to study the progression and pathophysiology of all forms of group II PH. With translation to PH as a result of left HF, with reduced or preserved ejection fraction, it can be both a drawback and an advantage that there is no actual left HF. On the one hand, this model could be missing circulating factors originating from left HF such as elevated natriuretic peptides and troponins (Gaggin & Januzzi, 2013), while on the other hand, we are able to study the isolated pulmonary vascular pathogenesis of CpcPH without influence of the left side of the heart. A limitation of this swine model, however, is that the conventional methods of calculating PVR ((mPAP − PCWP)/CO or (mPAP − LAP)/CO) do not result in true vascular resistance, as the resistance of the band around the lower pulmonary veins is also taken into account. Also the new parameter for differentiation between IpcPH and CpcPH, i.e. the diastolic pressure gradient, is not directly applicable in this model. In the present study, tPVRi (PAP/CI) was used to indicate changes in RV afterload as, with the current haemodynamic measurements, changes in vascular resistance of the upper and lower lobes cannot be distinguished from the changes in resistance induced by PVB.

### Oxygenation

The venous confluence drains both inferior (caudal) pulmonary lobes, which account for 80% of total lung mass in swine (Garcia‐Alvarez *et al*. 2013). As the animal grows while the band maintains a fixed venous stenosis diameter, blood flow to the lower lobes is gradually restricted, likely resulting in redistribution of the blood flow to the middle and upper pulmonary lobes. The increased flow to these lobes decreases capillary transit time, reflected by a reduced arterial $P_{O_{2}}$ and, to a lesser extent in arterial $S_{O_{2}}$, in PVB animals (Yuba, 1971). It could further be speculated that a decreased diffusion capacity in swine with PVB contributes to the decreased oxygenation at rest as is also observed in severe pulmonary arterial hypertension (PAH) and severe PH in HF (Trip *et al*. 2014; Hoeper *et al*. 2016). However, measurement of diffusion capacity requires respiratory mask‐testing which is technically challenging in swine and was not performed in the present study.

During exercise, the increase in cardiac output further decreases transit time, which resulted in decreased arterial $P_{O_{2}}$ and arterial O_2_ saturation in PVB swine. In exercising sham animals, arterial O_2_ saturation remained relatively constant, although arterial $P_{O_{2}}$ decreased slightly, but to a lesser extent than in swine with PVB. Tezosentan did not alter cardiac output, and arterial oxygenation. Importantly, the observation that arterial $P_{O_{2}}$ was unaffected by tezosentan infusion suggests that tezosentan did not result in pulmonary oedema.

### Pulmonary microvascular remodelling in PH: role of ET

Twelve weeks after induction of pulmonary vein banding, pulmonary small arteries demonstrated muscularization and thickening of the vascular wall, resulting in a relative reduction of the vascular lumen. This finding is consistent with the observation that pulmonary vascular remodelling in group II PH mainly consists of medial hypertrophy and muscularization of arterioles (Dickinson *et al*. 2013). Consistent with the increased muscularization, we found that maximal contraction of these vessels was also increased, which was accompanied by alterations in the ET pathway, a well‐known mediator of vascular remodelling (Shimoda *et al*. 2002; Shao *et al*. 2011; Shimoda & Laurie, 2013). It has been shown that the ET pathway is upregulated in PAH (Kawanabe & Nauli, 2011; Galie *et al*. 2016). Because of the abluminal release, circulating plasma ET levels are thought to be the result, at least in part, of spillover from the junctions between endothelial and smooth muscle cells and can therefore not be equated to ET activity in pathological states (Kawanabe & Nauli, 2011). However, plasma ET levels are upregulated in PAH and correlate with disease severity and haemodynamics (Stewart *et al*. 1991; Giaid *et al*. 1993).

In addition to increased ET levels, ET signalling may also be increased via upregulation of ET receptors. For example, ET_A_ receptors have been reported to be upregulated, while ET_B_ receptors may be up‐ or downregulated, depending on the type of PH, in clinical and experimental PH (Gosselin *et al*. 1997; Bauer *et al*. 2002; Sauvageau *et al*. 2009). We previously showed that in swine with PH secondary to myocardial infarction, plasma ET levels were increased, and infusion of exogenous ET resulted in a more pronounced pulmonary vasoconstriction compared to normal swine (Houweling *et al*. 2006). This increased response to ET appeared to be the result of an increased ET_A_‐mediated vasoconstriction. Also, plasma ET levels are increased in chronic HF, and correlate with the severity of symptoms and pulmonary haemodynamics (Cody *et al*. 1992; Pacher *et al*. 1996). This is accompanied by upregulation of ET_A_ and downregulation of ET_B_ receptors (Ponicke *et al*. 1998; Zolk *et al*. 1999; Staniloae *et al*. 2004).

The observation that ET_A_+ET_B_ blockade by tezosentan produced a more pronounced vasodilatation in awake PVB than sham animals confirms increased ET activity in type II PH *in vivo*. The present study showed an increased expression of PPET and ECE in lung tissue, and higher plasma ET levels in PVB as compared to sham at 10 and 12 weeks after PVB. As indicated in Fig. 4, two outliers were identified and removed from statistical analyses. Although this decreased the variation within groups, it did not alter statistical significance. The increased expression of PPET and ECE, as well as the increased plasma ET levels, is consistent with data that show that the lungs are the primary source of ET‐1 (Dupuis *et al*. 1996a,*b*), although the contribution of other organs cannot be excluded in the present study as PPET or ECE expression was only measured in lung tissue. Furthermore, it appeared that the plasma ET levels decreased over time in the sham‐operated swine, while these levels in PVB swine increased only slightly. Swine received the banding or sham surgery at the age of 3 weeks and were studied until the age of 16 weeks. The observation that in sham animals, plasma ET levels decreased over time is consistent with decreasing plasma ET levels over time in the first months after birth in humans (Yoshibayashi *et al*. 1991).

The higher plasma ET levels in PVB were accompanied by a decreased expression of ET_B_ receptors, which are responsible for ET clearance, in the lower lobes. In the present study, the ET_A_ receptor was the main receptor responsible for ET‐induced vasoconstriction, since ET_B_ receptor blockade did not affect this constriction in pulmonary small arteries from either sham or PVB animals. The maximal constriction to exogenous ET was increased, which likely reflects the increased muscularization of the pulmonary small arteries, as the response to KCl was similarly increased. Moreover, the normalized concentration response curve was shifted to the right in PVB pulmonary small arteries, suggesting desensitization of the pulmonary vasculature to ET. Although this desensitization was not accompanied by a decrease in ET_A_ receptor expression, it is possible that changes in pulmonary vascular ET_A_ receptor expression remained undetected as ET receptor expression was measured in bulk lung‐tissue expression, and hence expression in endothelial and smooth muscle cells cannot be distinguished from expression in bronchi and alveoli.

Interestingly, the vasodilator response to ET receptor blockade with tezosentan was blunted during exercise at 10 weeks, but not at 12 weeks, suggesting progressive activation of the ET system and/or suppression of ET‐mediated constriction during exercise. The latter is consistent with our previous findings that during exercise, nitric oxide blunts the vasoconstrictor effect of ET (Houweling *et al*. 2005). The attenuated vasodilatation to tezosentan during exercise at week 10 might thus be the result of an increase in nitric oxide during exercise that is no longer present at 12 weeks after PVB.

### Clinical relevance

Our study is the first to show that ET is upregulated in group II PH without an underlying disease that causes neurohumoral activation such as myocardial infarction or HF. The fact that an initially purely mechanical increase of pulmonary pressure and resistance induces over‐activation of the ET pathway suggests that the ET pathway might be an interesting target for therapy in this group of patients.

To date, a number of clinical trials have been performed with a variety of ET receptor antagonists (ERAs) in chronic HF, with generally rather disappointing results (Coletta & Cleland, 2001; Louis *et al*. 2001; Kalra *et al*. 2002; Luscher *et al*. 2002; Anand *et al*. 2004; Packer *et al*. 2005; McMurray *et al*. 2007; Koller *et al*. 2017; Vachiery *et al*. 2018). It should be noted, however, that all experimental ERAs were tested in the presence of conventional medical HF therapy, including angiotensin converting enzyme inhibitors, β‐blockers and angiotensin II and aldosterone receptor antagonists. It cannot be excluded that the added effect of ERAs was limited because of blunted neurohumoral activity by these other drugs. Indeed, in another clinical study, acute administration of darusentan produced a dose‐dependent change in CI, SVR, PVR, MAP, mPAP and PCWP when conventional medical therapy was interrupted (Spieker *et al*. 2000), whereas in the HEAT and EARTH trials, where medication was continued, darusentan produced no additional pulmonary haemodynamic effects (Luscher *et al*. 2002; Anand *et al*. 2004). Moreover, while in some clinical trials, ERAs did improve haemodynamic variables, including PCWP, PVR, SVR, CI and right atrial pressure (Louis *et al*. 2001; Luscher *et al*. 2002; McMurray *et al*. 2007), none resulted in improved outcome or clinical status. It is therefore important to note that in the present study the acute effect of ERAs were investigated, and that these results may not directly translate to positive long‐term studies. Future studies are thus required to investigate the pulmonary effects of chronic therapy with ERAs in group II PH.

The current guidelines of the European Society of Cardiology for treatment of group II PH recommend the treatment of the left ventricle, to optimize volume status, to take care of co‐morbidities and to be cautious in using pulmonary vasodilators (Galie *et al*. 2016). We believe a distinction must be made between group II PH as a result of left HF and non‐HF causes of group II PH. Non‐HF causes of group II PH include pulmonary vein stenosis, which can be either congenital or as a result of radiofrequency ablation in treatment of atrial fibrillation (Latson & Prieto, 2007; Manzar, 2007; Fender *et al*. 2016). Pulmonary vein stenosis causes a passive increase in PVR and mPAP, as was also shown in the present study. We also observed that the ET pathway is upregulated as early as 10 weeks after inducing the stenosis, and that ET contributes to an active increase of PVR and mPAP. Consequently, inhibiting the ET pathway could be beneficial in this group of patients to stop progression from IpcPH to CpcPH, especially because the most serious adverse effects in the clinical trials in HF patients (worsening of HF, peripheral/pulmonary oedema) might not apply to non‐HF patients. Recently, the US Food and Drug Administration approved the dual receptor antagonist bosentan for treatment of PH in children. While it will mainly be under investigation as a treatment of PAH, bosentan could also be utilized in treatment of children with PH as a result of congenital pulmonary vein stenosis. The observation in this study that ET‐induced vasoconstriction in isolated pulmonary small arteries appeared to be entirely dependent on ET_A_ receptors suggests ET_A_ blockade alone might be preferable to dual ERAs.

### Conclusions

Banding of the confluence of both inferior pulmonary veins in swine resulted in a progressive increase in pulmonary arterial pressure and resistance, which could be measured from week 5 until week 12 after banding, in the awake state, by chronic instrumentation. From week 10 onward, the pulmonary endothelin pathway was upregulated, likely contributing to pre‐capillary activation of the initially isolated post‐capillary pulmonary hypertension and leading to structural and functional vascular remodelling. Inhibition of the endothelin pathway could thus potentially provide a pharmacotherapeutic target for early stage post‐capillary pulmonary hypertension, especially in post‐capillary pulmonary hypertension with normal left heart function.

## Additional information

### Competing interests

No competing interests, financial or otherwise, are declared by the authors.

### Authors contributions

Study design: R.W.B.vD., A.G.A., B.I., I.R., D.J.D. and D.M. Performing experiments: R.W.B.vD., K.S. Z.C. and D.M. Data quality control and analysis: R.W.B.vD., K.S., Z.C., A.U., A.H.J.D., I.R., D.J.D. and D.M. Manuscript writing: R.W.B.vD. Manuscript revision: R.W.B.vD., K.S., Z.C., A.U., A.G.A., B.I., A.H.J.D., I.R., D.J.D. and D.M. All authors have read and approved the final version of this manuscript and agree to be accountable for all aspects of the work in ensuring that questions related to the accuracy or integrity of any part of the work are appropriately investigated and resolved. All persons designated as authors qualify for authorship, and all those who qualify for authorship are listed.

### Funding

This work was supported by the Netherlands Cardiovascular Research Initiative, the Dutch Heart Foundation, the Dutch Federation of University Medical Centres, the Netherlands Organization for Health Research and Development and the Royal Netherlands Academy of Science (CVON 2012‐08, Phaedra). This work was further supported by the Sophia Foundation for Medical Research (SSWO), The Netherlands, Grant S13‐12, 2012, and the China Scholarship Council (201606230252).
